# Associations between chronic obstructive pulmonary disease and ten common cancers: novel insights from Mendelian randomization analyses

**DOI:** 10.1186/s12885-024-12381-9

**Published:** 2024-05-17

**Authors:** Shixia Liao, Yanwen Wang, Jian Zhou, Yuting Liu, Shuangfei He, Lanying Zhang, Maomao Liu, Dongmei Wen, Pengpeng Sun, Guangbing Lu, Qi Wang, Yao Ouyang, Yongxiang Song

**Affiliations:** 1 Department of Respiratory and Critical Care Medicine, Affiliated Hospital of Zunyi Medical University, Guizhou, 563003 China; 2 West China School of Medicine, Sichuan University, Chengdu, 610041 China; 3 Department of Osteopathy, Affiliated Hospital of Zunyi Medical University, Guizhou, 563003 China; 4 Department of Respiration, Meishan Hospital of Traditional Chinese Medicine in Sichuan Province, Meishan, 620010 China; 5 China-Canada Medical and Health Science Association, Toronto, L3R 1A3 Canada; 6 Department of Thoracic Surgery, Affiliated Hospital of Zunyi Medical University, Guizhou, 563003 China

**Keywords:** COPD, Lung cancer, Bladder cancer, Common cancers, Mendelian randomization, Smoking

## Abstract

**Background:**

Chronic obstructive pulmonary disease (COPD) is a significant global health issue, suspected to elevate the risk for various cancers. This study sought to discern whether COPD serves as a risk marker or a causative factor for prevalent cancers.

**Methods:**

We employed univariable MR (UVMR) analyses to investigate the causal relationship between COPD and the top ten common cancers. Sensitivity analyses were performed to validate the main findings. Multivariable MR (MVMR) and two-step MR analyses were also conducted. False-discovery-rate (FDR) was used to correct multiple testing bias.

**Results:**

The UVMR analysis demonstrated notable associations between COPD and lung cancer (odds ratio [OR] = 1.42, 95%CI 1.15–1.77, FDR = 6.37 × 10^–3^). This relationship extends to lung cancer subtypes such as squamous cell carcinoma (LUSC), adenocarcinoma (LUAD), and small cell lung cancer (SCLC). A tentative link was also identified between COPD and bladder cancer (OR = 1.53, 95%CI 1.03–2.28, FDR = 0.125). No significant associations were found between COPD and other types of cancer. The MVMR analysis that adjusted for smoking, alcohol drinking, and body mass index did not identify any significant causal relationships between COPD and either lung or bladder cancer. However, the two-step MR analysis indicates that COPD mediated 19.2% (95% CI 12.7–26.1%), 36.1% (24.9–33.2%), 35.9% (25.7–34.9%), and 35.5% (26.2–34.8%) of the association between smoking and overall lung cancer, as well as LUAD, LUSC, and SCLC, respectively.

**Conclusions:**

COPD appears to act more as a risk marker than a direct cause of prevalent cancers. Importantly, it partially mediates the connection between smoking and lung cancer, underscoring its role in lung cancer prevention strategies.

**Supplementary Information:**

The online version contains supplementary material available at 10.1186/s12885-024-12381-9.

## Introduction

Chronic obstructive pulmonary disease (COPD) is a widespread global health issue, characterized by persistent airflow limitation and respiratory symptoms [1]. Its prevalence has been steadily increasing, resulting in a significant disease burden worldwide [2]. In addition to its well-established impact on lung function and overall health regardless of the smoking status, COPD has recently been identified as a potential risk factor for various types of cancer [3–7]. For example, findings from a Danish nationwide cohort study revealed that patients with first-time hospital-diagnosed COPD are at considerably increased risk of developing both lung cancer and extrapulmonary cancers including cancers of the larynx, tongue, oral cavity, pharynx, esophagus, stomach, liver, pancreas, cervix uteri, and urinary tract [4]. Likewise, a cohort study performed in Taiwanese showed that COPD patients had increased risk for both pulmonary and extrapulmonary cancers [7]. These studies have highlighted a systemic influence of COPD, potentially linked to factors such as chronic inflammation or oxidative stress associated with the disease [5, 8]. For instance, research indicates that COPD exacerbations are often accompanied by significant systemic oxidative stress [9], a factor that may contribute to carcinogenesis in organs beyond the lungs, such as the liver [10].

Although previous epidemiological studies have consistently demonstrated an association between COPD and increased cancer risk, it is important to acknowledge that observational studies are vulnerable to several methodological challenges. Reverse causality poses a notable concern, as the presence of cancer may influence the development or severity of COPD, rather than the other way around [11]. Furthermore, confounding variables, such as smoking, age, and comorbidities, can independently contribute to both COPD and cancer, potentially influencing the observed associations [12, 13]. The reliability and generalizability of findings may also be affected by the duration of follow-up and sample size limitations in these studies.

To address the limitations of observational studies and gain a deeper understanding of the causal relationship between COPD and cancer, alternative approaches are needed. Mendelian randomization (MR) analysis, utilizing genetic information as instrumental variables, offers a promising method for causal inference in observational studies [14, 15]. By leveraging genetic variants associated with COPD as proxies for the disease, MR analysis can help mitigate the effects of confounding and reverse causality, providing a more reliable estimate of the causal effect. For instance, using MR analysis, Higbee et al. found limited evidence for a causal effect of either lung function or COPD on Alzheimer's disease [16]. MR analysis performed by Zhu et al. showed a significant positive causal effect of COPD on resting heart rate [17]. However, there was yet no MR assessment for the association between COPD and common cancers.

In this study, we aimed to investigate the causal relationship between COPD and the top ten common cancers (i.e., cancers of lung, esophagus, liver, stomach, colon and rectum, thyroid, bladder, prostate, female breast, and cervix) [18] using MR analysis. Our findings hold the potential to enhance our understanding of the COPD-cancer link and have implications for targeted interventions, prevention strategies, and personalized treatment approaches in both COPD and cancer management.

## Materials and methods

### Study design

In this analysis, we constructed a two-sample MR analysis framework, in which COPD was set was the exposure and the ten common cancers were the outcomes. Genetic variants that associated with COPD were used as the instrumental variables (IVs). A valid IV for inferring causality between exposure and outcome should meet the following assumptions: (1) the IVs are associated with the exposure of interest; (2) there are no unmeasured confounders of the associations between IVs and outcome; and (3) the IVs affect the outcome only via the exposure of interest [19].

### GWAS summary data of instrumental variables of COPD

We retrieved genetic summary data of COPD from the Global Biobank Meta-analysis Initiative (GBMI), which incorporated 18 biobanks involving up to 1.8 million participants with diverse ancestries (Table 1) [20]. In this study, we only retrieved the genetic data of participants with European ancestry, of which 61,627 were diagnosed as COPD and 980,360 were defined as healthy controls (Supplementary Tables S1-2). Each biobank conducted genotyping, imputation and quality controls and estimated sample ancestry independently. Fixed-effect meta-analyses based on inverse-variance weighting were performed for COPD with all biobanks stratified by each ancestry and by sex. Thus, we also retrieved COPD GWAS summary data of males and females for sex-specific analysis (e.g., analysis for prostate and female breast cancers).

**Table 1:** GWAS sources of chronic obstructive pulmonary disease (COPD) and common cancers

Traits	PMID	Case (N)	Control (N)	Web source
COPD	36777996	61,627	980,360	http://results.globalbiobankmeta.org/
Lung cancer	28604730	85,716	29,266	GWAS-Catalog: GCST004748
Lung adenocarcinoma		66,756	11,273	GWAS-Catalog: GCST004744
Lung squamous cell carcinoma		63,053	7,426	GWAS-Catalog: GCST004750
Small cell lung carcinoma		24,108	2,664	GWAS-Catalog: GCST004746
Esophageal cancer^a^	27527254	10,279	17,159	GWAS-Catalog: GCST003740
Gastric cancer	FinnGen	1,054	238,678	https://storage.googleapis.com/finngen-public-data-r7/summary_stats/finngen_R7_C3_STOMACH_EXALLC.gz
Liver cancer	UK Biobank	539	419,992	https://pan.ukbb.broadinstitute.org/phenotypes/index.html
Colorectal cancer	UK Biobank	5,657	372,016	IEU-OpenGWAS: ieu-b-4965
Thyroid	36777996	6,997	1,369,273	http://results.globalbiobankmeta.org/
Prostate cancer	29892016	79,194	61,112	http://practical.icr.ac.uk/blog/?page_id=8164
Female breast cancer	32424353	133,384	113,789	https://bcac.ccge.medschl.cam.ac.uk/bcacdata/oncoarray/oncoarray-and-combined-summary-result/gwas-summary-associations-breast-cancer-risk-2020/
Bladder cancer	32887889	2,242	410,350	GWAS-Catalog: GCST90011817
Cervical cancer	32887889	6,563	410,350	GWAS-Catalog: GCST90011816

^a^including 6,167 European ancestry Barrett’s esophagus cases

We followed a series of quality control steps to identify eligible IVs for COPD. First, we extracted SNPs that showed an association with COPD at the traditional GWAS threshold (*P* < 5 × 10^–8^). Second, we performed a clumping process based on the linkage disequilibrium (LD) estimates from the European samples in the 1000 genomes project. We only retained the SNP that had the lower *P* value among those pairs of SNPs that had an LD estimate above the specified threshold (0.01) and a window size of 10,000 kb. Third, we removed SNPs with a minor allele frequency < 1%. We also calculated the F-statistics for the IVs of COPD [21]; a mean F-statistic > 10 denotes a low probability of weak-IV bias.

### GWAS summary data of common cancers

We retrieved the genetic summary data of ten common cancers from their respective GWAS (Table 1). When selecting GWAS for the common cancers, our primary criterion was the comprehensiveness and specificity of the data available for each cancer type. For certain cancers where specific GWAS were not available, we turned to large-scale databases such as FinnGen and the UK Biobank. Our choice was guided by the number of available cases, ensuring the robustness and reliability of our analysis. This strategy allowed us to leverage the most extensive datasets, thereby enhancing the validity of our findings and providing a comprehensive overview of the genetic associations across a broad spectrum of cancers. All cancer GWASs were limited to Europeans. For lung cancer, we also retrieved the GWAS summary data of its histological subtypes, that are lung adenocarcinoma (LUAD), lung squamous cell carcinoma (LUSC), and small cell lung carcinoma (SCLC).

Next, we extracted the statistics (i.e., beta coefficient and standard error) for the IVs from the cancer GWAS summary data and harmonized them with that of the COPD GWAS. If a requested SNP was not present in the cancer GWAS, we retrieved the data of an SNP proxy that had an LD estimate of *R*^2^ > 0.8 with the requested SNP. We corrected or directly excluded the effects of ambiguous SNPs with inconsistent alleles and palindromic SNPs with ambiguous strands in the subsequent two-sample MR analysis.

### Univariable Mendelian randomization analysis

We performed the univariable MR (UVMR) according to the following procedure. First, we tested for horizontal pleiotropy using the MR-PRESSO global test [22] and removed outliers (i.e., SNPs with *P* < 0.05) if horizontal pleiotropy was present. Second, we tested for between-SNP heterogeneity using the inverse variance weighting (IVW) method based on the SNPs that remained after pleiotropy correction. We used Cochran’s Q statistic to check for the presence of heterogeneity and removed SNPs with *P* < 1.00 in MR-PRESSO analysis if heterogeneity was significant (*P* value of Cochran’s Q statistic < 0.05). Third, we conducted MR analysis using the IVW method. We obtained the IVW estimate by meta-analyzing the SNP-specific Wald estimates using multiplicative random effects. We calculated the statistical power for MR analysis using mRnd website [23]. We also performed a series of sensitivity analyses using four different methods: MR-Egger regression, weighted median, weighted mode, and MRPRESSO methods. Additionally, we conducted a “leave-one-out” analysis to identify influential SNPs.

### Multivariable Mendelian randomization analysis

To further overcome the potential pleiotropy, we performed multivariable MR (MVMR) analysis [24], in which smoking, alcohol drinking, and body mass index (BMI) were included as the covariates. The genetic summary data of smoking and alcohol drinking were retrieved from a GWAS of risk tolerance and risky behaviors in over 1 million individuals [25]. Smoking was measured by its status (i.e., ever *vs*. never smokers), and alcohol drinking was measured by drinks per week. The genetic summary data of BMI was retrieved from a GWAS for height and BMI in ∼700000 individuals of European ancestry [26]. Multivariable weighted median method was applied if the presence of significant between-SNP heterogeneity, otherwise, multivariable IVW method was employed.

### Mediation analysis

Since smoking is a well-determined risk factor for both COPD and cancers, we performed a two-step MR analysis to investigate the mediation effect of COPD on the association between smoking and cancers [27]. To assess the indirect effect, we adopted the “product of coefficients” strategy. Two-step MR uses a series of UVMR analyses to estimate the total effect of the exposure on the outcome, the effect of the exposure on the mediator, and the effect of the mediator on the outcome [28]. The indirect effect of the exposure on the outcome can then be calculated by multiplying the effect of the exposure on the mediator and the effect of the mediator on the outcome. Standard errors were derived using the sum of squares method.

We used false-discovery rate (FDR) to adjust for multiple testing and an FDR < 0.05 was deemed statistically significant. A *P* value < 0.05 but FDR > 0.05 denotes a suggestive association. All statistics were performed using R program (v 4.1.1). *TwoSampleMR*, *MendelianRandomization*, and *MRPRESSO* packages were used for MR analyses.

## Results

Following rigorous quality control processes, we included a number of IVs, ranging from seven to 23, for MR analysis for cancers (Table 2; Supplementary Table S3). The mean F-statistics were all > 10, indicating a low probability of weak IV bias. However, significant between-SNP heterogeneities were observed for IVs in the MR analysis of lung cancer and its histological subtypes, bladder, colorectal, prostate, and cervical cancers (Table 2). No significant horizontal pleiotropy was found for any cancer type, further validating the robustness of the MR analysis. Thus, for MR analyses showing significant between-SNP heterogeneity, we reported estimates of weighted median method as the main findings, otherwise IVW estimates were reported. In the current scenario, we have enough statistical power to identify an association (represented by an odds ratio [OR]) less than 0.8 or greater than 1.2 between COPD and cancers. When aiming to identify an OR between 0.8 and 1.2, the statistical powers were decreased to varying degrees, ranging from 78 to 95.

**Table 2:** Mendelian randomization statistics for chronic obstructive pulmonary disease (COPD) and common cancers

Outcomes	No. of IV	F-statistics	Between-SNP heterogeneity			Horizontal pleiotropy			Statistical power to detect OR < 0.8 or > 1.2 (%)	Statistical power to detect OR between 0.8 and 1.2 (%)
			**Q-value**	***P* values**	**FDR**	**Egger-intercept**	***P* values**	**FDR**		
Lung cancer	16	174.8	40.7	**4.33 × 10**^**–6**^	**9.36 × 10**^**–4**^	-0.0099	0.548	0.854	100	82
Lung squamous cell carcinoma	19	199.5	116.6	**1.22 × 10**^**–17**^	**2.34 × 10**^**–15**^	-0.0875	0.011	0.098	100	95
Lung adenocarcinoma	18	182.6	123.5	**3.56 × 10**^**–9**^	**1.07 × 10**^**–7**^	-0.0664	0.057	0.133	100	93
Small cell lung carcinoma	20	201.5	81.7	**4.59 × 10**^**–11**^	**4.07 × 10**^**–9**^	-0.0917	0.052	0.133	100	93
Bladder cancer	22	246.5	36.9	**2.98 × 10**^**–4**^	**0.028**	-0.0687	0.024	0.098	100	93
Esophageal cancer	20	198.6	23.1	0.164	0.305	0.0029	0.678	0.962	100	92
Liver cancer	23	255.3	19.5	0.382	0.614	-0.0491	0.129	0.462	100	90
Colorectal cancer	22	246.5	39.7	**9.89 × 10**^**–5**^	**0.014**	0	0.710	0.962	100	93
Thyroid cancer	23	255.3	23.6	0.245	0.435	0.0213	0.110	0.387	100	90
Gastric cancer	22	246.5	28.4	0.062	0.186	0.0018	0.699	0.962	100	93
Prostate cancer	14	108.9	29.1	**7.59 × 10**^**–5**^	**0.013**	-0.0069	0.571	0.854	100	81
Cervical cancer	7	48.5	74.9	**8.55 × 10**^**–15**^	**2.60 × 10**^**–13**^	-0.1421	0.087	0.397	100	78
Female breast cancer	7	48.5	5.6	0.288	0.514	0.0180	0.550	0.854	100	78

### Findings of univariable Mendelian randomization analysis

**Fig. 1:**
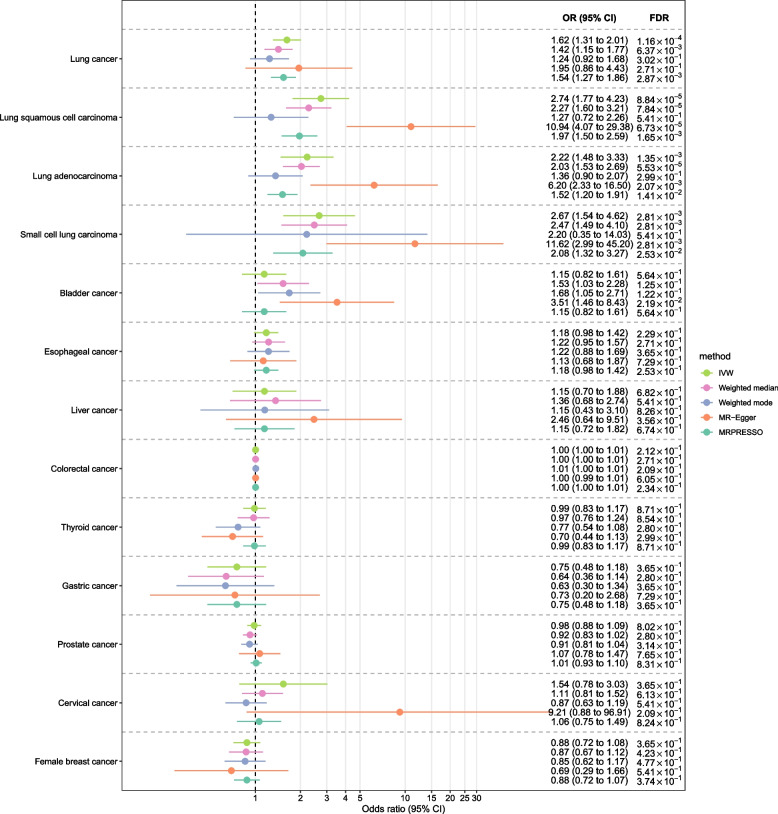
Genetic association between chronic obstructive pulmonary disease and common cancers according to univariable Mendelian randomization analysis

UVMR analysis (IVW method or weighted median method) suggested that COPD was significantly associated with the risk of lung cancer (OR = 1.42, 95% CI 1.15–1.77, FDR = 6.37 × 10^–3^), LUSC (OR = 2.27, 95% CI 1.60–3.21, FDR = 7.84 × 10^–5^), LUAD (OR = 2.03, 95% CI 1.53–2.69, FDR = 5.53 × 10^–5^), and SCLC (OR = 2.47, 95% CI 1.49–4.10, FDR = 2.81 × 10^–3^), and was suggestively associated with bladder cancer (OR = 1.53, 95% CI 1.03–2.28, FDR = 1.25 × 10^–1^) (Fig. 1). No significant or suggestive association was detected between COPD and other eight types of cancer.

**Fig. 2:**
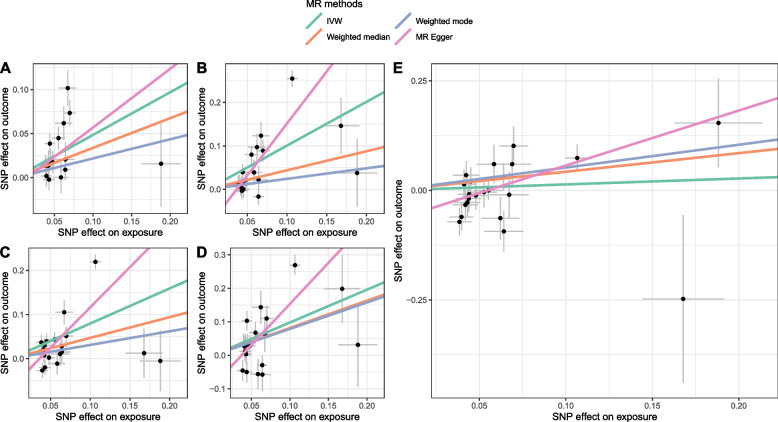
Scatter plot showing the SNP effects on both chronic obstructive pulmonary disease (exposure) and lung and bladder cancers (outcome). (The gray error bars denote the 95% confidence intervals of the effects; **A**-**E** represents lung cancer, lung squamous cell carcinoma, lung adenocarcinoma, small cell lung carcinoma, and bladder cancer, respectively.)

**Fig. 3:**
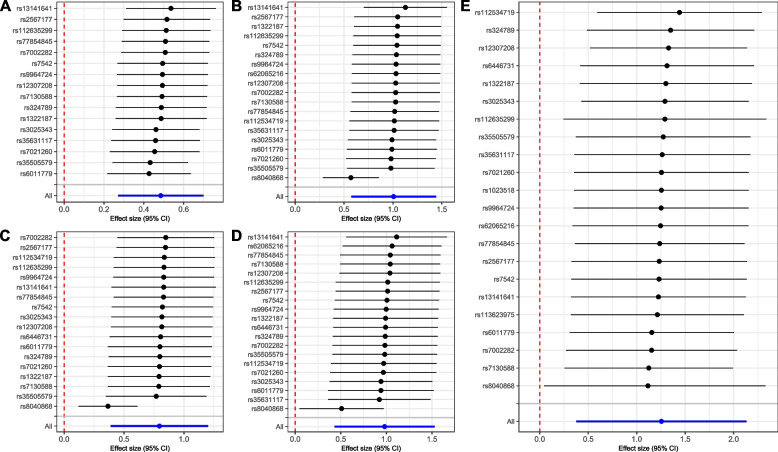
Leave-one-out analysis for chronic obstructive pulmonary disease and lung and bladder cancers. (The blue line denotes the integrated effect size; **A**-**E** represents lung cancer, lung squamous cell carcinoma, lung adenocarcinoma, small cell lung carcinoma, and bladder cancer, respectively)

For most COPD-cancer pairs, estimates from different MR methods were consistent in directions (Fig. 2; Supplementary Figure S1). For instance, all methods indicated a positive association between COPD and lung cancer while revealing a negative association between COPD and gastric cancer. Moreover, in the “leave-one-analysis”, we did not identify any potential outlier that significantly influences the association between COPD and cancers, namely the association did not significantly change when excluding any SNP (Fig. 3; Supplementary Figure S2).

### Findings of multivariable Mendelian randomization analysis

**Fig. 4:**
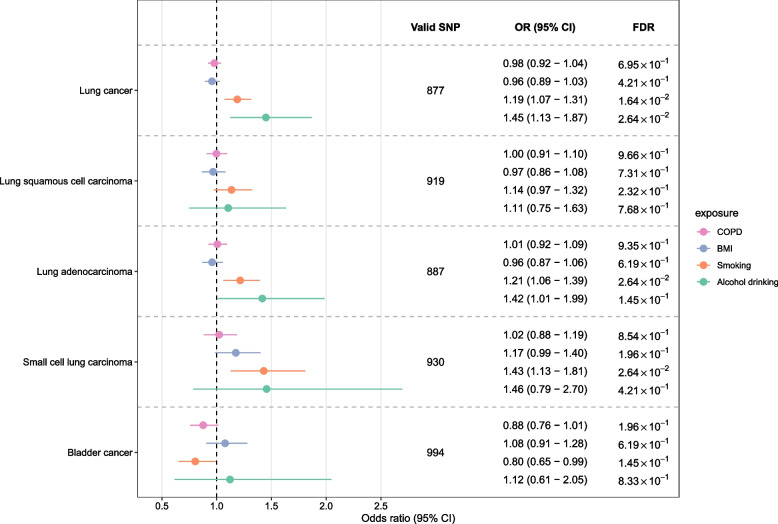
Genetic association between chronic obstructive pulmonary disease and lung and bladder cancers according to multivariable Mendelian randomization analysis

To further validate the significant and suggestive association that identified in UVMR analysis, we performed a MVMR for lung cancer (including its histological subtypes) and bladder cancer. A total of 877, 919, 887, 930, and 994 eligible IVs were used for these five types of cancer, respectively. MVMR analysis reported an OR of 0.98 (95% CI 0.92–1.04) for lung cancer, 1.00 (95% CI 0.91–1.10) for LUSC, 1.01 (95% CI 0.92–1.09) for LUAD, 1.02 (95% CI 0.88–1.19) for SCLC, and 0.88 (95% CI 0.76–1.01) for bladder cancer (Fig. 4). In this analysis, we found that smoking is a causal factor for lung cancer (OR = 1.19, 95% CI 1.07–1.31, FDR = 1.64 × 10^–2^) as well as its subtypes LUAD (OR = 1.21, 95% CI 1.06–1.39, FDR = 2.64 × 10^–2^) and SCLC (OR = 1.43, 95% CI 1.13–1.81, FDR = 2.64 × 10^–2^) (Fig. 4).

### Mediation effect of COPD

**Fig. 5:**
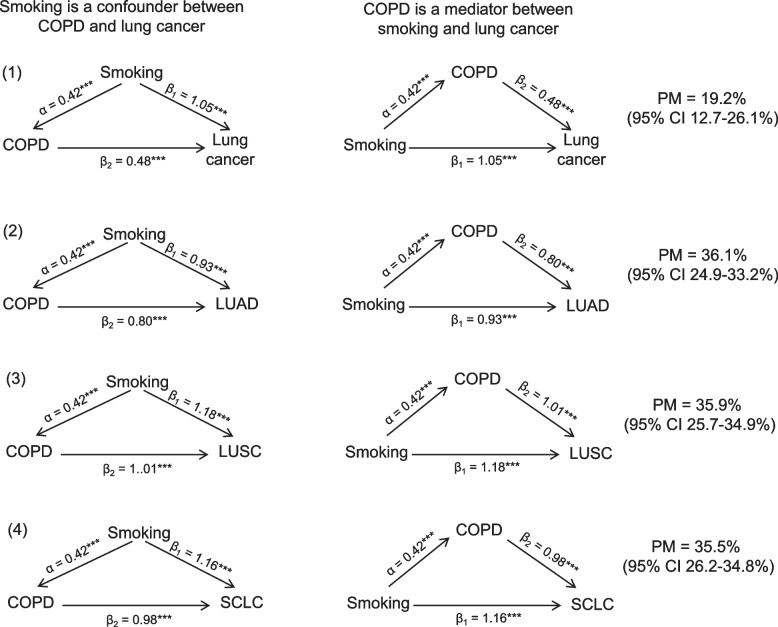
Directed acyclic graph representing the associations across smoking, COPD, and lung cancer. (α, β_1_, β_2_ were calculated from the univariable Mendelian randomization analysis. ***denotes *P* value < 0.001. PM means proportion mediated. LUAD, lung adenocarcinoma; LUSC, lung squamous cell carcinoma; SCLC, small cell lung carcinoma)

In our analysis, we focused on estimating the mediating effect of COPD in the relationship between smoking and lung cancer, given that there’s no causal link between smoking and bladder cancer risk (OR = 1.35, 95% CI 0.97–1.88). Through the UVMR analysis, a significant association was observed between smoking and both COPD and lung cancer (Fig. 5). This association was also present for different lung cancer subtypes. These results imply that while smoking acts as a confounder in the COPD-lung cancer relationship, COPD might also be a mediator for smoking’s effect on lung cancer. The two-step MR analysis indicates that COPD mediated 19.2% (95% CI 12.7–26.1%), 36.1% (24.9–33.2%), 35.9% (25.7–34.9%), and 35.5% (26.2–34.8%) of the association between smoking and overall lung cancer, as well as LUAD, LUSC, and SCLC subtypes, respectively (Fig. 5).

## Discussion

Our study aimed to investigate the causal relationship between COPD and common cancers using MR analysis. We comprehensively analyzed data for various cancer types, including lung, liver, gastric, esophageal, colorectal, thyroid, female breast, cervical, and prostate cancers. The results of our study reveal a significant causal association between COPD and lung cancer, specifically its histological subtypes, which include lung squamous cell carcinoma, lung adenocarcinoma, and small cell lung carcinoma. Additionally, we found evidence suggesting a potential causal association between COPD and bladder cancer. However, in multivariable MR analysis that included smoking, alcohol drinking, and BMI as the covariates, the associations between COPD and lung and bladder cancers become statistically non-significant. Our MR analysis did not detect any significant associations between COPD and the other cancer types investigated.

Observational studies have consistently reported an increased risk of lung cancer among individuals with COPD [29]. Although efforts have been made to adjust for common confounders such as smoking, studies investigating the association between COPD and cancer often encounter inherent limitations, including underadjustment for confounders and reverse causality, which can impact the interpretation of the observed associations. For example, the smoking data were typically obtained through questionnaires, making them susceptible to recall bias [30]. Moreover, smoking represents a complex and subjective variable that poses challenges in its precise measurement within epidemiological studies. The assessment of smoking varied among studies, with some utilizing a categorical approach by comparing never or former smokers to current smokers [31, 32], while others quantified smoking using pack-years [33, 34]. Given the inherent limitations, the findings of observational studies should be interpreted with cautions and further validations were also warranted. In the current study, by utilizing MR analysis, we aimed to overcome these limitations and provide robust evidence for the causal relationship between COPD and lung cancer and other common cancers. Our findings were largely aligning with previous research, strengthening the evidence for a causal link between COPD and lung cancer.

The underlying mechanisms driving the causal relationship between COPD and lung cancer can be explained by several factors. COPD is characterized by chronic inflammation and oxidative stress in the lungs, both of which play significant roles in the development and progression of lung cancer [35, 36]. Inflammatory mediators and reactive oxygen species generated in the lungs of COPD patients can lead to DNA damage, impaired DNA repair mechanisms, and disruption of cellular homeostasis [37]. These processes contribute to the oncogenic transformation observed in lung cancer. Furthermore, shared genetic factors and common environmental exposures, such as tobacco smoke, contribute to the co-occurrence of COPD and lung cancer [38, 39]. Genetic variations associated with COPD may also influence lung cancer risk through pleiotropic effects on cellular pathways involved in cancer development [40].

However, we have to note that the significantly positive association between COPD and lung cancer was disappeared when we further adjusted for their shared risk factors (i.e., smoking, alcohol drinking, and BMI). This finding suggested that the association between COPD and lung cancer may be confounded by smoking, namely COPD and lung cancer are sequelae of heavy smoking, although COPD always precedes lung cancer. In this case, COPD per se may not be a causer but only be a risk factor for lung cancer. We also found that COPD may be a mediator between smoking and lung cancer, suggesting a potential biological pathway: smoking leads to COPD, and then COPD, in turn, increases the risk of lung cancer. If COPD indeed acts as a mediator, it emphasizes the importance of early detection and management of COPD. By effectively managing and potentially slowing the progression of COPD, we might also reduce the risk of lung cancer among these patients. Interestingly, in our two-step MR analysis, it was observed that COPD mediated a higher percentage of the association between smoking and specific lung cancer subtypes (LUAD, LUSC, and SCLC) compared to overall lung cancer. This finding could be partly explained by the differential impact of genetic variants, which serve as instrumental variables in our analysis, on these specific subtypes versus lung cancer in general. It underscores the nuanced role that genetic factors may play in the mediation effects across different cancer subtypes, a reflection of the complex interplay between genetics, COPD, and lung cancer pathogenesis.

Regarding the potential causal association between COPD and bladder cancer, our UVMR findings reveal a suggestive relationship, although this association was become non-significant in MVMR analysis. The association between COPD and bladder cancer was rarely reported in observational studies. Chronic inflammation in the respiratory tract of COPD patients can induce systemic inflammation, which may contribute to the development of bladder cancer. Inflammatory mediators and other inflammatory markers released in response to COPD-related inflammation can potentially affect the bladder's cellular environment and promote carcinogenesis [41]. Furthermore, shared risk factors, such as smoking and occupational exposures, may contribute to the observed association between COPD and bladder cancer [41, 42]. Smoking, in particular, is a well-established risk factor for both COPD and bladder cancer and likely plays a substantial role in the shared pathogenesis of these conditions [43].

While our study provides valuable insights into the causal relationship between COPD and lung cancer and suggests a potential association with bladder cancer, it is important to acknowledge the limitations. MR analysis relies on several assumptions, including the validity of genetic instruments and the absence of pleiotropy. Although we employed robust methods to minimize biases associated with these assumptions, some residual confounding or pleiotropy may still be present. Additionally, our study focused on a selected set of common cancers, and we did not explore associations with other cancer types. Therefore, caution should be exercised when extrapolating our findings to all cancer types. Furthermore, the generalizability of our results may be limited to populations with similar ethnic backgrounds as the cohorts used for MR analysis. Finally, while our study employed comprehensive strategies to minimize the impact of pleiotropy, such as rigorous SNP selection and advanced sensitivity analyses, we recognize the inherent challenges in completely ruling out pleiotropy in MR studies [44]. For instance, SNPs associated with COPD may also be involved in biological pathways such as inflammation, oxidative stress, and DNA damage, all of which are implicated in the development of various cancers. This overlap highlights the complexity of genetic influences in disease processes and underscores the need for cautious interpretation of MR findings.

## Conclusion

In conclusion, our study provides evidence for a causal association between COPD and lung cancer. We also found indications of a potential causal association between COPD and bladder cancer. However, these associations may be confounded by smoking. Further research is warranted to validate our findings, elucidate the underlying mechanisms, explore associations with other cancer types, and assess the potential impact of interventions.

### Supplementary Information

Supplementary Material 1.

## Data Availability

No datasets were generated or analysed during the current study.

## Abbreviations

COPD: Chronic obstructive pulmonary disease
MR: Mendelian Randomization
UVMR: Univariable Mendelian randomization
MVMR: Multivariable Mendelian randomization
GWAS: Genome-wide association study
IVW: Inverse variance weighted
FDR: False-discovery-rate
IV: Instrumental variable
LUAD: Lung adenocarcinoma
LUSC: Lung squamous cell carcinoma
SCLC: Small cell lung carcinoma

## References

[1] Christenson SA, Smith BM, Bafadhel M, Putcha N (2022). Chronic obstructive pulmonary disease. Lancet (London, England).

[2] Adeloye D, Song P, Zhu Y, Campbell H, Sheikh A, Rudan I (2022). Global, regional, and national prevalence of, and risk factors for, chronic obstructive pulmonary disease (COPD) in 2019: a systematic review and modelling analysis. Lancet Respir Med.

[3] Ante Z, Ernst P, Brassard P (2022). Risk of gastric cancer in chronic obstructive pulmonary disease. Eur J Cancer Prev.

[4] Kornum JB, Sværke C, Thomsen RW, Lange P, Sørensen HT (2012). Chronic obstructive pulmonary disease and cancer risk: a Danish nationwide cohort study. Respir Med.

[5] Ahn SV, Lee E, Park B, Jung JH, Park JE, Sheen SS, Park KJ, Hwang SC, Park JB, Park HS (2020). Cancer development in patients with COPD: a retrospective analysis of the National Health Insurance Service-National Sample Cohort in Korea. BMC Pulm Med.

[6] Hsu WL, Chen HY, Chang FW, Hsu RJ (2019). Does chronic obstructive pulmonary disease increase the risk of prostate cancer? A nationwide population-based study. Int J Chron Obstruct Pulmon Dis.

[7] Chiang CL, Hu YW, Wu CH, Chen YT, Liu CJ, Luo YH, Chen YM, Chen TJ, Su KC, Chou KT (2016). Spectrum of cancer risk among Taiwanese with chronic obstructive pulmonary disease. Int J Clin Oncol.

[8] MacNee W (2013). Systemic inflammatory biomarkers and co-morbidities of chronic obstructive pulmonary disease. Ann Med.

[9] Wiegman CH, Li F, Ryffel B, Togbe D, Chung KF (1957). Oxidative stress in ozone-induced chronic lung inflammation and emphysema: a facet of chronic obstructive pulmonary disease. Front Immunol.

[10] Banerjee P, Gaddam N, Chandler V, Chakraborty S (2023). Oxidative stress-induced liver damage and remodeling of the liver vasculature. Am J Pathol.

[11] Godtfredsen NS, Lam TH, Hansel TT, Leon ME, Gray N, Dresler C, Burns DM, Prescott E, Vestbo J (2008). COPD-related morbidity and mortality after smoking cessation: status of the evidence. Eur Respir J.

[12] Kridin K, Comaneshter D, Batat E, Cohen AD (2018). COPD and lung cancer in patients with pemphigus- a population based study. Respir Med.

[13] Lederer DJ, Bell SC, Branson RD, Chalmers JD, Marshall R, Maslove DM, Ost DE, Punjabi NM, Schatz M, Smyth AR (2019). Control of confounding and reporting of results in causal inference studies. Guidance for authors from editors of respiratory, sleep, and critical care journals. Ann Am Thorac Soc.

[14] Davey Smith G, Hemani G (2014). Mendelian randomization: genetic anchors for causal inference in epidemiological studies. Hum Mol Genet.

[15] Sekula P, Del Greco MF, Pattaro C, Köttgen A (2016). Mendelian randomization as an approach to assess causality using observational data. J Am Soc Nephrol.

[16] Higbee D, Granell R, Walton E, Korologou-Linden R, Davey Smith G, Dodd J (2021). Examining the possible causal relationship between lung function, COPD and Alzheimer's disease: a Mendelian randomisation study. BMJ Open Respir Res..

[17] Zhu Z, Wang X, Li X, Lin Y, Shen S, Liu CL, Hobbs BD, Hasegawa K, Liang L, Boezen HM (2019). Genetic overlap of chronic obstructive pulmonary disease and cardiovascular disease-related traits: a large-scale genome-wide cross-trait analysis. Respir Res.

[18] Sung H, Ferlay J, Siegel RL, Laversanne M, Soerjomataram I, Jemal A, Bray F (2021). Global cancer statistics 2020: GLOBOCAN estimates of incidence and mortality worldwide for 36 cancers in 185 countries. CA Cancer J Clin..

[19] Davies NM, Holmes MV, Davey Smith G (2018). Reading Mendelian randomisation studies: a guide, glossary, and checklist for clinicians. BMJ (Clinical research ed).

[20] Zhou W, Kanai M, Wu KH, Rasheed H, Tsuo K, Hirbo JB, Wang Y, Bhattacharya A, Zhao H, Namba S (2022). Global Biobank Meta-analysis Initiative: Powering genetic discovery across human disease. Cell Genom.

[21] Jiang H, Hu D, Wang J, Zhang B, He C, Ning J (2022). Adiponectin and the risk of gastrointestinal cancers in East Asians: Mendelian randomization analysis. Cancer Med.

[22] Verbanck M, Chen CY, Neale B, Do R (2018). Detection of widespread horizontal pleiotropy in causal relationships inferred from Mendelian randomization between complex traits and diseases. Nat Genet.

[23] Brion MJ, Shakhbazov K, Visscher PM (2013). Calculating statistical power in Mendelian randomization studies. Int J Epidemiol.

[24] Burgess S, Thompson SG (2015). Multivariable Mendelian randomization: the use of pleiotropic genetic variants to estimate causal effects. Am J Epidemiol.

[25] Karlsson Linnér R, Biroli P, Kong E, Meddens SFW, Wedow R, Fontana MA, Lebreton M, Tino SP, Abdellaoui A, Hammerschlag AR (2019). Genome-wide association analyses of risk tolerance and risky behaviors in over 1 million individuals identify hundreds of loci and shared genetic influences. Nat Genet.

[26] Yengo L, Sidorenko J, Kemper KE, Zheng Z, Wood AR, Weedon MN, Frayling TM, Hirschhorn J, Yang J, Visscher PM (2018). Meta-analysis of genome-wide association studies for height and body mass index in ∼700000 individuals of European ancestry. Hum Mol Genet.

[27] Carter AR, Sanderson E, Hammerton G, Richmond RC, Davey Smith G, Heron J, Taylor AE, Davies NM, Howe LD (2021). Mendelian randomisation for mediation analysis: current methods and challenges for implementation. Eur J Epidemiol.

[28] Sanderson E (2021). Multivariable Mendelian Randomization and Mediation. Cold Spring Harb Perspect Med.

[29] Park HY, Kang D, Shin SH, Yoo KH, Rhee CK, Suh GY, Kim H, Shim YM, Guallar E, Cho J (2020). Chronic obstructive pulmonary disease and lung cancer incidence in never smokers: a cohort study. Thorax.

[30] Coughlin SS (1990). Recall bias in epidemiologic studies. J Clin Epidemiol.

[31] Wheaton AG, Liu Y, Croft JB, VanFrank B, Croxton TL, Punturieri A, Postow L, Greenlund KJ (2019). Chronic obstructive pulmonary disease and smoking status - United States, 2017. MMWR Morb Mortal Wkly Rep.

[32] Li G, Wang H, Wang K, Wang W, Dong F, Qian Y, Gong H, Hui C, Xu G, Li Y (2017). The association between smoking and blood pressure in men: a cross-sectional study. BMC Public Health.

[33] Peto J (2012). That the effects of smoking should be measured in pack-years: misconceptions 4. Br J Cancer.

[34] Conlon MS, Johnson KC, Bewick MA, Lafrenie RM, Donner A (2010). Smoking (active and passive), N-acetyltransferase 2, and risk of breast cancer. Cancer Epidemiol.

[35] Yao H, Rahman I (2009). Current concepts on the role of inflammation in COPD and lung cancer. Curr Opin Pharmacol.

[36] Adcock IM, Caramori G, Barnes PJ (2011). Chronic obstructive pulmonary disease and lung cancer: new molecular insights. Respiration.

[37] Rosanna DP, Salvatore C (2012). Reactive oxygen species, inflammation, and lung diseases. Curr Pharm Des.

[38] Atchley WT, Alvarez C, Saxena-Beem S, Schwartz TA, Ishizawar RC, Patel KP, Rivera MP (2021). Immune checkpoint inhibitor-related pneumonitis in lung cancer: real-world incidence, risk factors, and management practices across six health care centers in North Carolina. Chest.

[39] Caramori G, Ruggeri P, Mumby S, Ieni A, Lo Bello F, Chimankar V, Donovan C, Andò F, Nucera F, Coppolino I (2019). Molecular links between COPD and lung cancer: new targets for drug discovery?. Expert Opin Ther Targets.

[40] Yuan ST, Ellingrod VL, Schipper M, Stringer KA, Cai X, Hayman JA, Yu J, Lawrence TS, Kong FM (2013). Genetic variations in TGFβ1, tPA, and ACE and radiation-induced thoracic toxicities in patients with non-small-cell lung cancer. J Thorac Oncol.

[41] Milara J, Cortijo J (2012). Tobacco, inflammation, and respiratory tract cancer. Curr Pharm Des.

[42] Rava M, Czachorowski MJ, Silverman D, Márquez M, Kishore S, Tardón A, Serra C, García-Closas M, Garcia-Closas R, Carrato A (2018). Asthma status is associated with decreased risk of aggressive urothelial bladder cancer. Int J Cancer.

[43] Samanic C, Kogevinas M, Dosemeci M, Malats N, Real FX, Garcia-Closas M, Serra C, Carrato A, García-Closas R, Sala M (2006). Smoking and bladder cancer in Spain: effects of tobacco type, timing, environmental tobacco smoke, and gender. Cancer Epidemiol Biomarkers Prev.

[44] Yang Q, Sanderson E, Tilling K, Borges MC, Lawlor DA (2022). Exploring and mitigating potential bias when genetic instrumental variables are associated with multiple non-exposure traits in Mendelian randomization. Eur J Epidemiol.

