# Supplementary material for: Associations between chronic obstructive pulmonary disease and ten common cancers: novel insights from Mendelian randomization analyses
Source: BMC Cancer. 2024 May 17;24:601. doi: 10.1186/s12885-024-12381-9 (PMC11100175; doi:10.1186/s12885-024-12381-9)
Supplement: Supplementary file 1 — Supplementary Material 1. [file 12885_2024_12381_MOESM1_ESM.docx]

**Supplementary materials for**

**Associations between chronic obstructive pulmonary disease and ten common cancers: novel insights from Mendelian randomization analyses**

Liao et al.

Figure S1. Scatter plot showing the SNP effects on both chronic obstructive pulmonary disease (exposure) and other types of cancer (outcome).

(The gray error bars denote the 95% confidence intervals of the effects; A-H represents cancer of esophagus, liver, colon and rectum, thyroid, stomach, prostate, cervix, and female breast, respectively.)


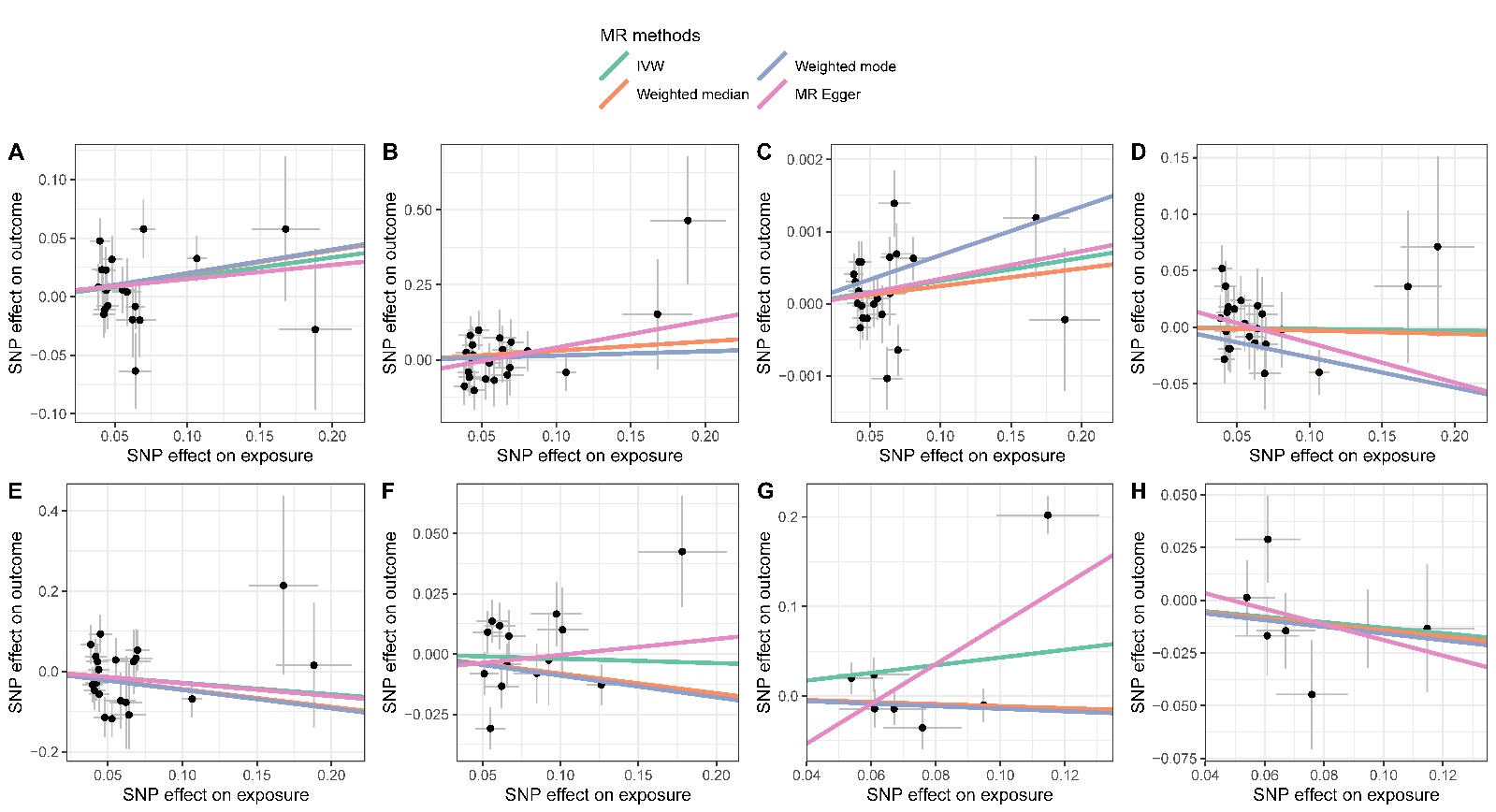


Figure S2. Leave-one-out analysis for chronic obstructive pulmonary disease and other types of cancer.

(The blue line denotes the integrated effect size; A-H represents cancer of esophagus, liver, colon and rectum, thyroid, stomach, prostate, cervix, and female breast, respectively.


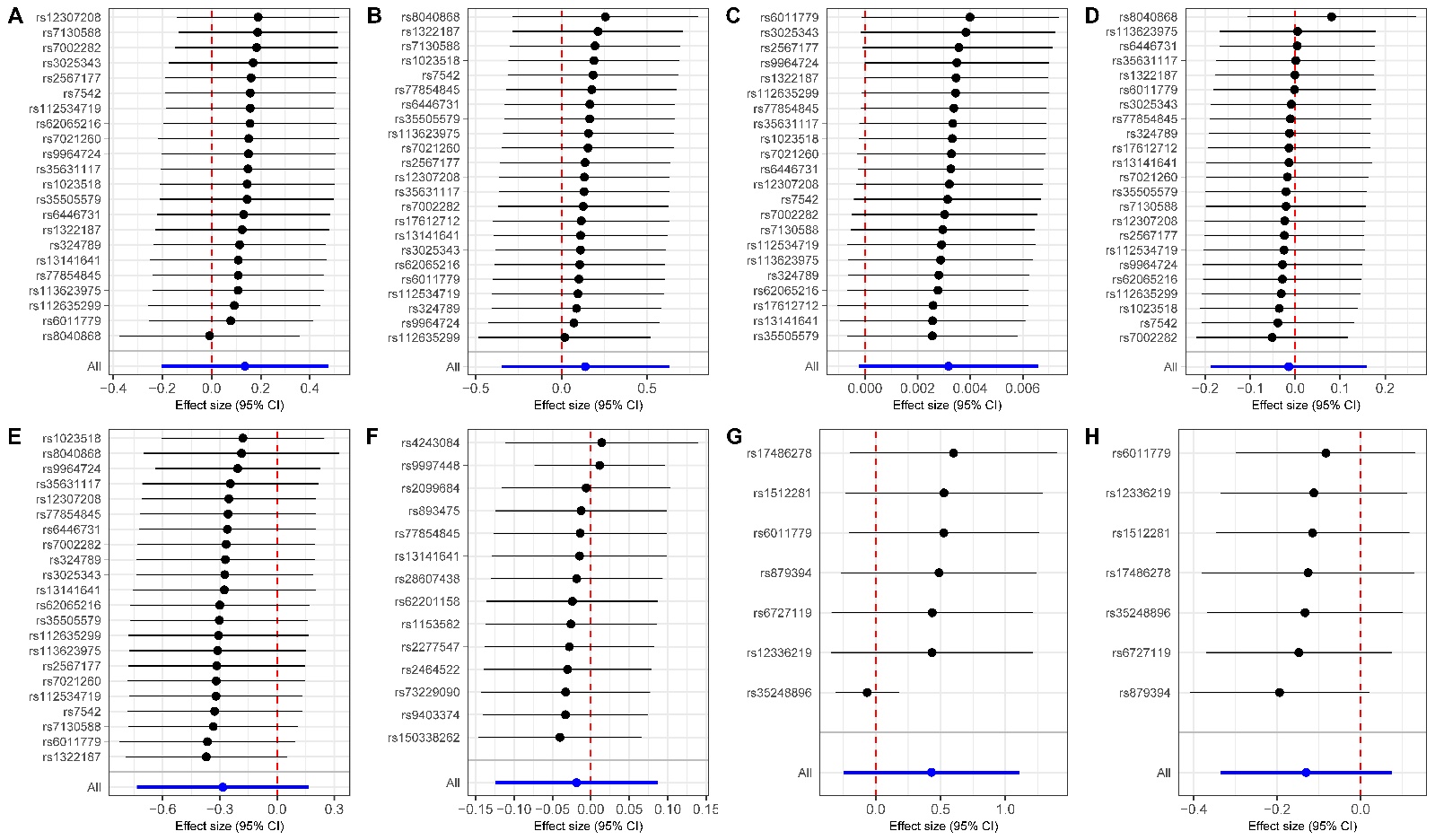


Table S1. Sample size for COPD GWAS in the Global Biobank Meta-analysis Initiative.

| Endpoint | Sex | Ancestry | Biobank | Case | Control | Stage |
| --- | --- | --- | --- | --- | --- | --- |
| COPD | Both sex | non-Finnish European | BioMe | 611 | 8193 | discovery |
| COPD | Both sex | non-Finnish European | BioVU | 4962 | 55358 | discovery |
| COPD | Both sex | non-Finnish European | CCPM | 1533 | 18365 | discovery |
| COPD | Both sex | non-Finnish European | ESTBB | 10596 | 115610 | discovery |
| COPD | Both sex | Finnish | FinnGen | 6915 | 186723 | discovery |
| COPD | Both sex | non-Finnish European | GS | 239 | 19793 | discovery |
| COPD | Both sex | non-Finnish European | HUNT | 5529 | 61332 | discovery |
| COPD | Both sex | non-Finnish European | Lifelines | 3103 | 22372 | discovery |
| COPD | Both sex | non-Finnish European | MGB | 3922 | 18033 | discovery |
| COPD | Both sex | non-Finnish European | MGI | 4834 | 37287 | discovery |
| COPD | Both sex | non-Finnish European | UCLA | 2005 | 14636 | discovery |
| COPD | Both sex | non-Finnish European | UKBB | 14310 | 379656 | discovery |
| COPD | Male | non-Finnish European | BioMe | 313 | 3906 | discovery |
| COPD | Male | non-Finnish European | BioVU | 2631 | 24203 | discovery |
| COPD | Male | non-Finnish European | CCPM | 769 | 7181 | discovery |
| COPD | Male | Finnish | FinnGen | 4521 | 81410 | discovery |
| COPD | Male | non-Finnish European | GS | 95 | 8132 | discovery |
| COPD | Male | non-Finnish European | HUNT | 2868 | 28653 | discovery |
| COPD | Male | non-Finnish European | Lifelines | 1582 | 8902 | discovery |
| COPD | Male | non-Finnish European | MGI | 2471 | 18580 | discovery |
| COPD | Male | non-Finnish European | UKBB | 7663 | 176589 | discovery |
| COPD | Female | non-Finnish European | BioMe | 298 | 4287 | discovery |
| COPD | Female | non-Finnish European | BioVU | 2359 | 31155 | discovery |
| COPD | Female | non-Finnish European | CCPM | 764 | 11184 | discovery |
| COPD | Female | Finnish | FinnGen | 2394 | 105313 | discovery |
| COPD | Female | non-Finnish European | GS | 144 | 11661 | discovery |
| COPD | Female | non-Finnish European | HUNT | 2661 | 32679 | discovery |
| COPD | Female | non-Finnish European | Lifelines | 1521 | 13470 | discovery |
| COPD | Female | non-Finnish European | MGI | 2363 | 18707 | discovery |
| COPD | Female | non-Finnish European | UKBB | 6758 | 206058 | discovery |
| COPD | Both sex | Europeans | CanPath | 137 | 7123 | replication |
| COPD | Both sex | Europeans | bbofa | 273 | 12440 | replication |
| COPD | Female | Europeans | bbofa | 214 | 8873 | replication |
| COPD | Male | Europeans | bbofa | 59 | 3567 | replication |
| COPD | Both sex | Europeans | PMBB | 2658 | 23439 | replication |
| COPD | Female | Europeans | PMBB | 947 | 10371 | replication |
| COPD | Male | Europeans | PMBB | 1711 | 13068 | replication |

Table S2. Phenotype definitions recommended by GBMI.

| **COPD Cases** | **Controls** |
| --- | --- |
| 491,491.0,491.1,491.2,491.20,491.21,491.22,491.8,491.9,492,492.0,492.8,494,494.0,494.1,496, J41,J41.0,J41.1,J41.8,J42,J43,J43.0,J43.1,J43.2,J43.8,J43.9,J44,J44.0,J44.1,J44.8,J44.9,J47,J44.1,J44,J44.0,J47.1,J47.9,J47.0,J47,J43.2,J43.0,J43.1,J43.8,J43.9,J43,J41.8,J42,J41.1,J41.0,J41,J44.9 | Subjects with the following ICD codes were excluding from controls: 490,493,493.0,493.00,493.01,493.02,493.1,493.10,493.11,493.12,493.2,493.20,493.21,493.22,493.8,493.81,493.82,493.9,493.90,493.91,493.92,519.11,J40,J45,J45.0,J45.1,J45.8,J45.9,J46,J45.31,J45.901,J45.42,J45.41,J45.51,J45.990,J45.902,J45.21,J45.52,J45.32,J45.22,J40,J45.909,J45.998,J45.40,J45.20,J45,J45.991,J45.90,J45.30,J45.50,J98.01 |

Table S3. Instrumental variables for COPD.

| **Exposure** | **CHR** | **Position** | **Other allele.** | **Effect allele.** | **SNP** | **EAF** | **Beta** | **SE** | **P value** |
| --- | --- | --- | --- | --- | --- | --- | --- | --- | --- |
| COPD | 4 | 3283024 | G | A | rs6446731 | 0.2942 | 0.041153 | 0.007281 | 1.58E-08 |
|  | 4 | 66938586 | G | T | rs35631117 | 0.5407 | -0.04412 | 0.006699 | 4.54E-11 |
|  | 4 | 111450477 | T | C | rs77854845 | 0.1316 | -0.05849 | 0.010049 | 5.88E-09 |
|  | 4 | 144585304 | T | C | rs13141641 | 0.4527 | -0.06407 | 0.006769 | 2.92E-21 |
|  | 5 | 132458080 | G | T | rs1023518 | 0.284 | 0.052684 | 0.007462 | 1.66E-12 |
|  | 6 | 32648168 | A | T | rs17612712 | 0.4094 | 0.080828 | 0.012309 | 5.15E-11 |
|  | 6 | 164951975 | G | T | rs1322187 | 0.3253 | 0.045065 | 0.007881 | 1.08E-08 |
|  | 7 | 117456239 | T | C | rs112534719 | 0.02358 | 0.16781 | 0.023421 | 7.78E-13 |
|  | 8 | 10809483 | A | G | rs7002282 | 0.5831 | -0.03969 | 0.006987 | 1.34E-08 |
|  | 8 | 27479528 | A | T | rs35505579 | 0.09941 | 0.067262 | 0.011123 | 1.47E-09 |
|  | 8 | 27555088 | A | G | rs113623975 | 0.1188 | -0.06895 | 0.010938 | 2.91E-10 |
|  | 9 | 125908872 | C | T | rs7021260 | 0.7144 | 0.055274 | 0.008413 | 5.02E-11 |
|  | 9 | 133613233 | G | A | rs3025343 | 0.09592 | 0.062135 | 0.011071 | 2.00E-08 |
|  | 11 | 76559639 | A | G | rs7130588 | 0.3342 | 0.038492 | 0.007048 | 4.73E-08 |
|  | 12 | 67723961 | T | C | rs12307208 | 0.09872 | -0.06417 | 0.011339 | 1.52E-08 |
|  | 12 | 83553708 | G | T | rs324789 | 0.3877 | -0.04238 | 0.007747 | 4.49E-08 |
|  | 14 | 94371805 | G | T | rs112635299 | 0.02004 | 0.18822 | 0.025347 | 1.12E-13 |
|  | 15 | 78618839 | T | C | rs8040868 | 0.3877 | 0.10658 | 0.006838 | 9.00E-55 |
|  | 16 | 30114519 | G | C | rs7542 | 0.4444 | -0.0419 | 0.007379 | 1.36E-08 |
|  | 17 | 40062520 | G | A | rs62065216 | 0.4436 | 0.04386 | 0.007131 | 7.71E-10 |
|  | 18 | 37579161 | C | T | rs9964724 | 0.7011 | -0.04799 | 0.007295 | 4.77E-11 |
|  | 18 | 40870233 | A | C | rs2567177 | 0.3617 | 0.043086 | 0.007483 | 8.53E-09 |
|  | 20 | 63352965 | C | T | rs6011779 | 0.7938 | -0.06982 | 0.008321 | 4.83E-17 |
| COPD in males | 1 | 161530340 | A | G | rs2099684 | 0.2941 | 0.062192 | 0.010968 | 1.42E-08 |
|  | 2 | 228688181 | A | G | rs62201158 | 0.07839 | -0.10131 | 0.016468 | 7.66E-10 |
|  | 3 | 25501784 | C | T | rs1153582 | 0.2142 | -0.06671 | 0.010777 | 6.00E-10 |
|  | 4 | 7851332 | C | A | rs28607438 | 0.1603 | 0.092395 | 0.013895 | 2.94E-11 |
|  | 4 | 88939692 | G | A | rs2464522 | 0.7188 | 0.060897 | 0.00987 | 6.83E-10 |
|  | 4 | 111450477 | T | C | rs77854845 | 0.1333 | -0.08461 | 0.015006 | 1.71E-08 |
|  | 4 | 139949361 | C | T | rs9997448 | 0.337 | -0.05496 | 0.009774 | 1.88E-08 |
|  | 4 | 144585304 | T | C | rs13141641 | 0.4092 | -0.06542 | 0.009005 | 3.74E-13 |
|  | 5 | 132464267 | C | T | rs11748326 | 0.2069 | 0.066458 | 0.011003 | 1.54E-09 |
|  | 5 | 148476959 | G | A | rs7733410 | 0.4935 | -0.05647 | 0.009425 | 2.08E-09 |
|  | 6 | 32005780 | T | C | rs150338262 | 0.1234 | -0.1781 | 0.028475 | 3.99E-10 |
|  | 6 | 142241727 | C | T | rs9403374 | 0.4296 | -0.056 | 0.010262 | 4.85E-08 |
|  | 8 | 27584610 | C | A | rs73229090 | 0.1069 | -0.0973 | 0.016385 | 2.88E-09 |
|  | 12 | 111823834 | G | A | rs11525583 | 0.1701 | 0.093615 | 0.016906 | 3.07E-08 |
|  | 14 | 94371805 | G | T | rs112635299 | 0.02047 | 0.22876 | 0.038672 | 3.31E-09 |
|  | 15 | 67177870 | A | G | rs893475 | 0.3784 | -0.05099 | 0.009333 | 4.67E-08 |
|  | 15 | 78619330 | G | C | rs4243084 | 0.3232 | 0.12609 | 0.009335 | 1.42E-41 |
|  | 15 | 78790089 | A | G | rs2277547 | 0.2879 | -0.05317 | 0.009663 | 3.75E-08 |
|  | 20 | 63352965 | C | T | rs6011779 | 0.7987 | -0.07255 | 0.012876 | 1.76E-08 |
| COPD in females | 2 | 59295660 | A | G | rs6727119 | 0.4364 | -0.05392 | 0.009703 | 2.74E-08 |
|  | 3 | 168992055 | G | T | rs879394 | 0.2593 | 0.061017 | 0.011019 | 3.07E-08 |
|  | 4 | 144513749 | A | G | rs1512281 | 0.4114 | -0.06705 | 0.009955 | 1.63E-11 |
|  | 6 | 32639593 | C | T | rs35248896 | 0.2392 | 0.11467 | 0.015987 | 7.35E-13 |
|  | 9 | 125969996 | C | T | rs12336219 | 0.6798 | 0.060822 | 0.010867 | 2.18E-08 |
|  | 15 | 78575140 | A | C | rs17486278 | 0.3212 | 0.094802 | 0.010246 | 2.19E-20 |
|  | 20 | 63352965 | C | T | rs6011779 | 0.7964 | -0.07583 | 0.01213 | 4.06E-10 |
